# Rosuvastatin Synergistically Enhances the Antinociceptive Efficacy of Duloxetine in Paclitaxel-Induced Neuropathic Pain in Mice

**DOI:** 10.3390/ijms24098359

**Published:** 2023-05-06

**Authors:** Nicolás Lobos, Sebastián Lux, Ramiro Javier Zepeda, Teresa Pelissier, José Luis Marcos, Gonzalo Bustos-Quevedo, Alejandro Hernández, Luis Constandil

**Affiliations:** 1 Laboratory of Neurobiology, Department of Biology, Faculty of Chemistry and Biology, University of Santiago de Chile, Santiago 9170022, Chile; nicolasloboszambrano@gmail.com (N.L.); sebastianluxfebre@gmail.com (S.L.); terepelissier@gmail.com (T.P.); gonzalo.bustos@usach.cl (G.B.-Q.); alejandro.hernandez@usach.cl (A.H.); 2 Critical Care Unit, Barros Luco Trudeau Hospital, Santiago 8900085, Chile; 3 Department of Neuroscience, Faculty of Medicine, University of Chile, Santiago 8380453, Chile; rzepeda@ciq.uchile.cl; 4 Escuela de Ciencias Agrícolas y Veterinarias, Universidad Viña del Mar, Viña del Mar 2572007, Chile; drjmarcos@gmail.com; 5 Center for the Development of Nanoscience and Nanotechnology (CEDENNA), Santiago 9170124, Chile

**Keywords:** rosuvastatin, duloxetine, paclitaxel, chronic pain, chemotherapy-induced neuropathy, isobolographic study

## Abstract

Paclitaxel, a widely used cancer chemotherapeutic agent, has high incidence of neurotoxicity associated with the production of neuropathic pain, for which only duloxetine has shown significant but moderate analgesic effect. Since statins, classically used to reduce hypercholesterolemia, have shown antinociceptive effect in preclinical studies on neuropathic pain, we studied whether the antinociceptive efficacy of duloxetine could be synergistically potentiated by rosuvastatin in a model of paclitaxel-induced neuropathy in mice. The astrocytic and microglial responses in the spinal cord of paclitaxel-treated mice were also assessed by measuring GFAP and CD11b proteins, respectively. Paclitaxel treatment did not impair motor coordination and balance in rotarod testing. Rosuvastatin, duloxetine, and the rosuvastatin/duloxetine combination (combined at equieffective doses) dose-dependently decreased mechanical allodynia (ED_30_, von Frey testing) and thermal hyperalgesia (ED_50_, hot plate testing) in paclitaxel-treated mice. Isobolographic analysis showed a superadditive interaction for rosuvastatin and duloxetine, as both the ED_30_ and ED_50_ for the rosuvastatin/duloxetine combination contained only a quarter of each drug compared to the individual drugs. The rosuvastatin/duloxetine combination reversed paclitaxel-induced GFAP overexpression, indicating that such effects might depend in part on astrocyte inactivation. Results suggest that statins could be useful in synergistically enhancing the efficacy of duloxetine in some chemotherapy-induced neuropathic conditions.

## 1. Introduction

Cancer is the second leading cause of death globally and is responsible for nearly 10 million deaths in 2020 [1]. Complications associated with its treatment are an emerging problem for the clinician, especially chemotherapy-induced peripheral neuropathy (CIPN), which affects more than one-third of patients [2,3]. CIPN is a particularly disabling pathology for patients that is difficult to manage, which can even lead to a decrease in or even suspension of the antineoplastic treatment. Characteristic symptoms of CIPN include allodynia and hyperalgesia, as in other neuropathic pain. In allodynia, the milder tactile sensory stimulus causes pain, while in hyperalgesia, the stimulation that produces typically mild pain, such as pinching or pressure, generates severe pain.

The mechanisms by which antineoplastic drugs cause neuropathic pain are complex, with numerous modifications found in primary sensory neurons and in dorsal horn cells in rodent models of CIPN, such as changes in ion channels (increase in sodium channels Nav1.7, decrease in potassium channels Kir1.1 and Kir3.4, increase in calcium channels Cav3.2) and in transient receptor potential channels (increase in TRPV1 channel), together with deficits in mitochondrial function and changes in immune cell interactions (monocyte/macrophage infiltration and microglial activation), with concurrent high levels of pro-inflammatory cytokines (for recent reviews see [4,5]).

The current treatment of CIPN relies on two main approaches: prevention (dose adjustment of the antineoplastic drug, administration of neuroprotective-like agents), and drug-based symptomatic treatment [6]. Among the various agents that have been examined for the prevention (chemoprotectants, anticonvulsants, antidepressants, vitamins, minerals, dietary supplements) and/or management (opioids, tri-cyclic anti-depressants, gabapentin, and topical gel with either baclofen, amitriptyline, or ketamine) of CIPN, only duloxetine has shown a clear (but moderate) effect [4,7], becoming the only treatment recommendation agreed upon by the American Society of Clinical Oncology [8]. Duloxetine is a balanced serotonin and noradrenaline reuptake inhibitor, and there is good evidence to support the use of dual serotonin and noradrenaline inhibitors in the management of neuropathic pain complaints [9]. Indeed, noradrenaline and serotonin are key target neurotransmitters in the suppression of painful response to peripheral nociceptive stimuli, by inhibiting the transmission of nociceptive information at the level of the spinal synapses between nociceptive afferents and dorsal horn neurons [10]. Unfortunately, today it seems clear that there are neither established preventive nor symptomatic treatments for CIPN aside from of duloxetine [11], and therefore CIPN remains a prominent clinical problem for patients receiving common cytotoxic chemotherapy regimens. Therefore, the corollary is that more preclinical research is needed on drugs with potential translational clinical value that can target the molecular mechanisms underlying chemotherapy-associated peripheral neuropathy.

It has recently been reported that rosuvastatin, a synthetically made hydrophilic statin that undergoes low first-pass extraction in the liver [12], produces thermal and mechanical antinociception in a model of neuropathy induced in mice by the antineoplastic drug paclitaxel [13]. This is in line with previous reports showing an association between the anti-inflammatory and antinociceptive effects of statins in mice models of acute pain [14,15]. Furthermore, it is also known that statins can attenuate peripheral neuropathy in different animal models, such as diabetic neuropathy [16], chronic constriction injury [17,18,19,20], and ischemia reperfusion nerve injury [21,22]. In close accordance, 4-week atorvastatin treatment was found to produce a significant reduction in the level of pain in patients with spinal cord injury, but only six months after atorvastatin administration [23]. In the paclitaxel-induced neuropathy mouse model, together with the generation of antinociception, it was found that the rosuvastatin treatment reversed the increased levels of IL-1β and of lipid peroxidation in the spinal cord of these animals, while attenuating the decreases in the ratio of reduced/oxidized glutathione [13], thus suggesting a central antinociceptive site of action for rosuvastatin, possibly on spinal glial cells. These observations open the possibility of testing whether statins could synergistically enhance the antinociceptive activity of duloxetine in rodent models of CIPN, since both types of drugs appear to act by different central mechanisms—one of the prerequisites for suspecting superadditive effects of a combination of drugs [24]. As is well-known, the combination of two (or more) different drugs that exert a similar effect on a particular symptom (e.g., analgesic effect), but produce differing side effects due to a distinct mechanism of action, constitutes a useful strategy to increase the desired effect while maintaining a low level of unwanted side effects.

Therefore, in the present preclinical study, we set out to compare the antinociceptive therapeutic effects of rosuvastatin and duloxetine on mechanical allodynia and thermal hyperalgesia in a mouse model of paclitaxel-induced neuropathy, and to examine by isobolographic analysis whether the antinociceptive effects of these two drugs can interact synergistically when combined in equieffective doses. Intraperitoneal paclitaxel administration under different dosages and protocols has shown to induce sensory neuropathy in rats and mice, with hyperalgesic/allodynic effects that are largely dose-independent [13,25,26,27]. In addition, to study the involvement of spinal astrocytes and/or microglia in antinociception induced by treatment with rosuvastatin and duloxetine, the levels of glial fibrillary acidic protein (GFAP), a marker of astrocytes, and CD11b protein, a marker of microglia, were measured in the spinal dorsal horn of neuropathic mice treated with equieffective rosuvastatin/duloxetine combinations or vehicle alone.

## 2. Results

### 2.1. Algesimetric Evaluation and Motor Performance in Mice with Paclitaxel-Induced Neuropathic Pain

**Figure 1:**
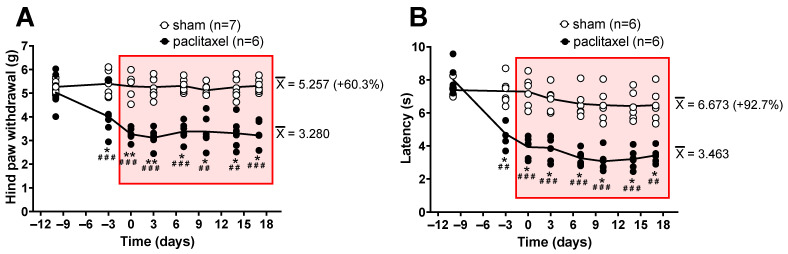
Paclitaxel 2 mg/kg i.p. (6 mice) or vehicle (6 mice) was administered as i.p. injections for 5 consecutive days, starting from day −10. (**A**) Time course of the allodynic effect induced by paclitaxel, as revealed by reduction in the hindlimb withdrawal threshold (in g) upon electronic von Frey testing. Intragroup statistics: * *p* < 0.05, ** *p* < 0.01, repeated measures two-way ANOVA, F_time_ (3.316, 36.48) = 6.697, compared scores after paclitaxel against pre-injection basal scores at day −10. Intergroup statistics: ^##^ *p* < 0.01, ^###^ *p* < 0.001, repeated measures two-way ANOVA, F_treatment_ F (1, 10) = 172.4, compared scores after paclitaxel against corresponding scores obtained in sham mice. (**B**) Time course of the hyperalgesic effect induced by paclitaxel, as revealed by reduction in the response latency (in s) upon hot plate testing. Intragroup statistics: * *p* < 0.05, repeated measures two-way ANOVA, F_time_ (4.076, 40.76) = 46.77, compared scores after paclitaxel against pre-injection basal scores at day −10. Intergroup statistics: ^##^ *p* < 0.01, ^###^ *p* < 0.001, repeated measures two-way ANOVA, F_treatment_ (1, 10) = 62.33, compared scores after paclitaxel against corresponding scores obtained in sham mice. In (**A**,**B**), the mean of scores of sham and paclitaxel-treated mice between day 0 and day 17 (inside the red square) are shown at the right side of the figure (in parentheses is shown how much higher, in percentage, is the mean of scores in sham versus paclitaxel-treated mice).

Paclitaxel-induced neuropathic pain was assessed through the production of mechanical allodynia and heat hyperalgesia using the electronic von Frey and the hot plate algesimetric tests, respectively. In the electronic von Frey test, mice receiving 2 mg/kg paclitaxel developed significant mechanical allodynia starting from day 7 after the first administration of paclitaxel, reaching quite stable allodynic scores from day 10 with respect to both the baseline pretreatment measurement and the sham group. Allodynia remained significant until day 17, where von Frey testing was stopped (Figure 1A). In the hot plate, the paclitaxel 2 mg/kg group showed significant heat hyperalgesia starting from day 7 after the first administration of paclitaxel, showing rather stable hyperalgesic scores at day 10 with respect to both the pretreatment measurement and the sham group. Hyperalgesia remained significant until day 17, where hot plate testing was stopped (Figure 1B).

**Figure 2:**
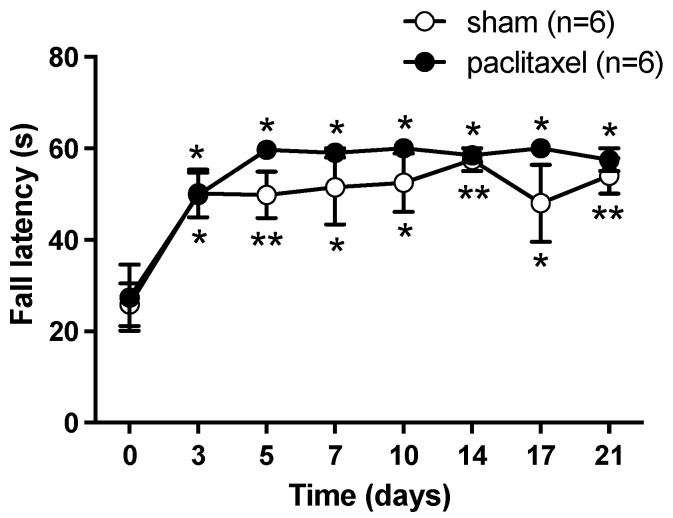
Effect of 2 mg/kg paclitaxel on motor coordination and balance, as related to vehicle, in rotarod testing. Paclitaxel or vehicle was administered as i.p. injections for 5 consecutive days, starting from day 0. Values are means ± SEM of 6 mice per group. Intergroup statistics: not significant, repeated measures two-way ANOVA, F_treatment_ (1, 10) = 1.018, scores after paclitaxel against corresponding scores in sham mice. Intragroup statistics: * *p* < 0.05, ** *p* < 0.01, repeated measures two-way ANOVA, F_time_ (2.512, 25.12) = 21.54, scores after paclitaxel or vehicle against pre-injection basal scores at day 0.

In addition, in order to rule out motor effects possibly induced by paclitaxel, which could alter the nociceptive response of mice to algesimetric testing, the rotarod test was performed. The 2 mg/kg paclitaxel group did not differ statistically from the vehicle group with respect to motor coordination and balance, and both groups of mice similarly improved motor performance scores over time as compared with pre-paclitaxel scores, indicating a similar learning ability of motor skills (Figure 2).

### 2.2. Antinociceptive Effect of Rosuvastatine, Duloxetine, or Their Combination on Paclitaxel-Induced Neuropathic Pain, as Measured in the Electronic von Frey Test

**Figure 3:**
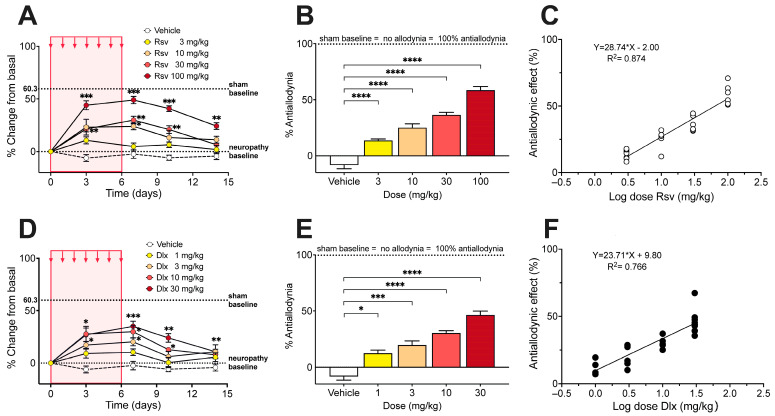
Effect of rosuvastatin and duloxetine on mechanical allodynia induced by paclitaxel in mice. (**A**) Time course of the antiallodynic effect of 7 injection (red area) of vehicle and 3, 10, 30, and 100 mg/kg i.p. rosuvastatin (Rsv), expressed as % change of electronic von Frey scores (in g) regarding basal score at day 0 (i.e., 10 days after starting paclitaxel administration). Intragroup statistics: * *p* < 0.05, ** *p* < 0.01, *** *p* < 0.001, two-way ANOVA, F_time_ (3.423, 82.14) = 44.86. (**B**) % Antiallodynia after vehicle and 3, 10, 30, and 100 mg/kg i.p. rosuvastatin, calculated from area under curves of (**A**), considering 100% antiallodynia (i.e., no allodynia) the sham control baseline (taken from Figure 1A). Statistics: **** *p* < 0.0001, one-way ANOVA, F (4, 22) = 68.36. (**C**) Dose-response data representing the antiallodynic effect (in %) of rosuvastatin (Rsv), expressed as dose logarithm. The equation for linear regression and the goodness of fit (R^2^) is shown in the graph. The ED_30_ of rosuvastatin was calculated from the regression line. (**D**) Time course of the antiallodynic effect of vehicle and 1, 3, 10, and 30 mg/kg i.p. duloxetine, expressed as % change of electronic von Frey scores (in g) regarding basal score at day 0 (i.e., 10 days after starting paclitaxel administration). Intragroup statistics: * *p* < 0.05, ** *p* < 0.01, *** *p* < 0.001, two-way ANOVA, F_time_ (2.963, 65.18) = 25.31. (**E**) % Antiallodynia after vehicle and 1, 3, 10, and 30 mg/kg i.p. duloxetine, calculated from area under curves of (**D**), considering 100% antiallodynia (i.e., no allodynia) the sham control baseline (taken from Figure 1A). Statistics: * *p* < 0.05, *** *p* < 0.001, **** *p* < 0.0001, one-way ANOVA, F (4, 22) = 32.92. (**F**) Dose-response data representing the antiallodynic effect (in %) of duloxetine (Dlx), expressed as dose logarithm. The equation for linear regression and the goodness of fit (R^2^) is shown in the graph. The ED_30_ of duloxetine was calculated from the regression line. In (**A**,**B**,**D**,**E**), values are means ± SEM. In all panels n = 6 mice per group.

Once mechanical allodynia had fully developed (day 10 after the first of five consecutive i.p. injections of paclitaxel), i.p. administration of rosuvastatin during 7 consecutive days induced a dose-dependent increase in the withdrawal threshold in the right hindlimb, expressed as % change of the basal score before rosuvastatin (Figure 3A). The % Antiallodynia during the whole 14 days of testing, calculated from the area under curves, amounted to 13.6 ± 1.4%, 25.0 ± 3.4%, 36.4 ± 2.3%, and 58.5 ± 3.1%, for doses of 3, 10, 30, and 100 mg/kg of rosuvastatin, respectively, all the % Antiallodynia scores being significantly higher than that obtained after vehicle administration (Figure 3B). The calculated ED_30_ value for rosuvastatin was 12.98 mg/kg, with a 95% confidence interval (95% CI) of 10.36 mg/kg to 16.26 mg/kg (Figure 3C).

Under a similar administration protocol than that used with rosuvastatine, i.p. duloxetine during 7 consecutive days induced a dose-dependent increase in the withdrawal threshold in the right hindlimb (Figure 3D). The % Antiallodynia during the whole 14 days of testing amounted to 12.2 ± 2.8%, 19.6 ± 3.5%, 30.4 ± 1.9%, and 46.4 ± 3.3%, for doses of 1, 3, 10, and 30 mg/kg of duloxetine, respectively, the % Antiallodynia scores being significantly higher than that obtained after vehicle administration (Figure 3E). The calculated ED_30_ value for duloxetine was 7.10 mg/kg with a 95% CI of 5.06 mg/kg to 9.97 mg/kg (Figure 3F).

**Figure 4:**
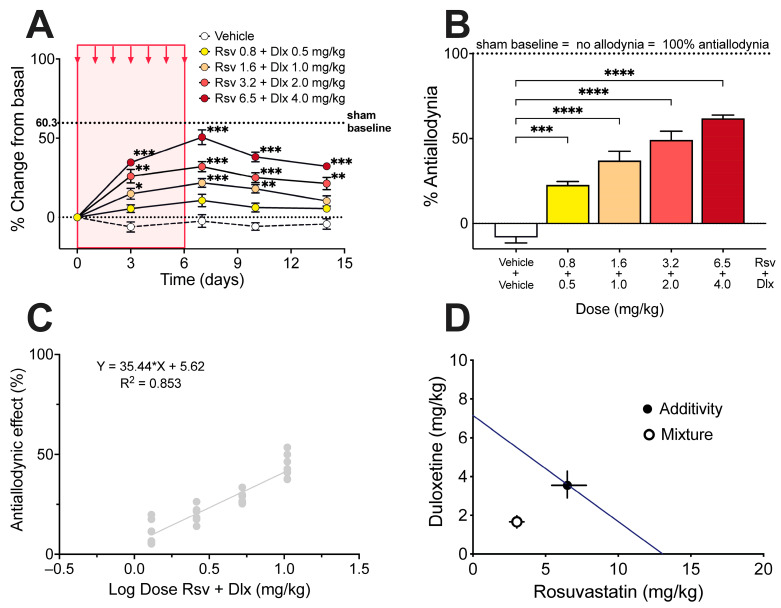
Effect of rosuvastatin/duloxetine combination on mechanical allodynia induced by paclitaxel in mice, and isobologram of the interaction. (**A**) Time course of the antiallodynic effect of vehicle and rosuvastatin (Rsv)/duloxetine (Dlx) combination administered in fixed ratios of their respective ED_30_, expressed as % change of electronic von Frey scores (in g) regarding basal score at day 0 (i.e., 10 days after starting paclitaxel administration). Intragroup statistics: * *p* < 0.05, ** *p* < 0.01, *** *p* < 0.001, two-way ANOVA, F_time_ (3.293, 92.21) = 72.97. (**B**) % Antiallodynia after vehicle and rosuvastatin/duloxetine combination, calculated from area under curves of (**A**), considering 100% antiallodynia (i.e., no allodynia) the sham control baseline (taken from Figure 1A). Statistics: *** *p* < 0.001, **** *p* < 0.0001, one-way ANOVA, F (4, 22) = 30.22. (**C**) Dose-response data representing the antiallodynic effect (in %) of rosuvastatin (Rsv)/duloxetine (Dlx) combination, expressed as dose logarithm. The equation for linear regression and the goodness of fit (R^2^) is shown in the graph. The ED_30_ of rosuvastatin/duloxetine combination was calculated from the regression line. (**D**) Isobologram of interaction for the antiallodynic effect of the rosuvastatin/duloxetine combination in mice with paclitaxel-induced neuropathy. The black circle on the straight line represents the point of theoretical additivity of the combination, whereas the white circle corresponds to the experimental point. The experimental point was significantly different from the theoretical point (mean ± SEM, *p* < 0.001), with an interaction index γ = 0.466, indicating superadditive interaction. The standard errors for rosuvastatin and duloxetine are resolved into rosuvastatin (abscissa scale) and duloxetine (ordinate scale) components and shown by horizontal and vertical bars, respectively.

From the ED_30_ for both rosuvastatin and duloxetine, it was possible to calculate the theoretical ED_30_ of the combination with a 95% confidence interval, and to establish equieffective submultiples of them by mixing the drugs at a fixed 1:1 ratio of their respective ED_30_, (1/2, 1/4, 1/8, and 1/16). The administration of the combination of rosuvastatin and duloxetine, in equieffective proportions of their respective ED_30_, induced a dose-dependent increase in the withdrawal threshold in the right hindlimb (Figure 4A). The % Antiallodynia for the total 14-day period of testing showed that the combination of rosuvastatin and duloxetine in fixed ratios of 1/2, 1/4, 1/8, and 1/16 of their respective ED_30_ produced % Antiallodynia scores of 22.6 ± 1.9%, 36.9 ± 5.5%, 49.1 ± 5.0%, and 61.8 ± 1.9%, respectively. All these antiallodynia scores were significantly higher than those obtained under vehicle administration (Figure 4B).

The experimental ED_30_ for the rosuvastatin/duloxetine association upon mechanical allodynia measured in the von Frey test was 4.67 mg/kg with 95% CI from 4.05 mg/kg to 5.40 mg/kg (Figure 4C), which can be decomposed in 3.02 mg/kg rosuvastatin plus 1.65 mg/kg duloxetine. Isobolographic analysis for the administration of rosuvastatin/duloxetine combination showed that the experimental ED_30_ was significantly lower than the theoretical additive ED_30_, with an interaction index γ = 0.466, which means a superadditive effect of the combination upon paclitaxel-induced mechanical allodynia (Figure 4D).

### 2.3. Antinociceptive Effect of Rosuvastatine, Duloxetine, or Their Combination on Paclitaxel-Induced Neuropathic Pain, as Measured in the Hot Plate Test

**Figure 5:**
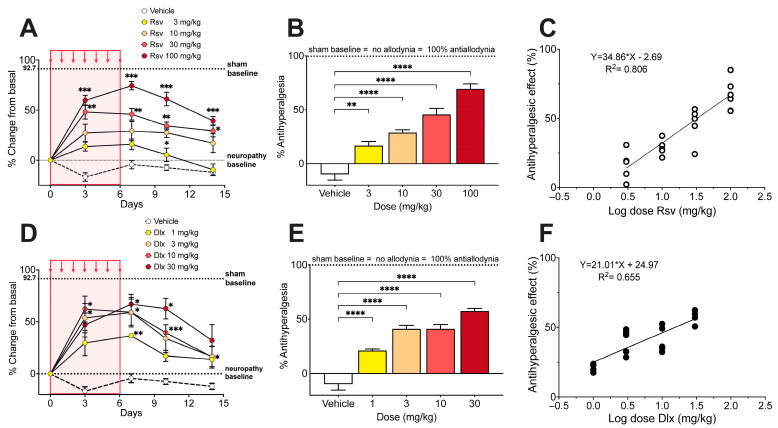
Effect of rosuvastatin and duloxetine on thermal hyperalgesia induced by paclitaxel in mice. (**A**) Time course of the antihyperalgesic effect of vehicle and 3, 10, 30, and 100 mg/kg i.p. rosuvastatin (Rsv), expressed as % change of hot plate scores (in s) regarding basal score at day 0 (i.e., 10 days after starting paclitaxel administration). Intragroup statistics: * *p* < 0.05, ** *p* < 0.01, *** *p* < 0.001, two-way ANOVA, F_time_ (3.170, 72.91) = 46.70. (**B**) % Antihyperalgesia after vehicle and 3, 10, 30, and 100 mg/kg i.p. rosuvastatin, calculated from area under curves of (**A**), considering 100% antihyperalgesia (i.e., no hyperalgesia) the sham control baseline (taken from Figure 1B). Statistics: ** *p* < 0.01, **** *p* < 0.0001, one-way ANOVA, F (4, 22) = 42.26. (**C**) Dose-response data representing the antihyperalgesic effect (in %) of rosuvastatin (Rsv), expressed as dose logarithm. The equation for linear regression and the goodness of fit (R^2^) is shown in the graph. The ED_50_ of rosuvastatin was calculated from the regression line. (**D**) Time course of the antihyperalgesic effect of vehicle and 1, 3, 10, and 30 mg/kg i.p. duloxetine (Dlx), expressed as % change of hot plate scores regarding basal score at day 0 (i.e., 10 days after starting paclitaxel administration). Intragroup statistics: * *p* < 0.05, ** *p* < 0.01, *** *p* < 0.001, two-way ANOVA, F_time_ (3.329, 59.93) = 33.70. (**E**) % Antihyperalgesia after vehicle and 1, 3, 10, and 30 mg/kg i.p. duloxetine, calculated from area under curves of (**D**), considering 100% antihyperalgesia (i.e., no hyperalgesia) the sham control baseline (taken from Figure 1B). Statistics: **** *p* < 0.0001, one-way ANOVA, F (4, 22) = 43.41. (**F**) Dose-response data representing the antihyperalgesic effect (in %) of duloxetine (Dlx), expressed as dose logarithm. The equation for linear regression and the goodness of fit (R^2^) is shown in the graph. The ED_50_ of duloxetine was calculated from the regression line. In (**A**,**B**,**D**,**E**), values are means ± SEM. In all panels n = 6 mice per group.

Algesimetric testing of rosuvastatin, duloxetine, or their combination started 10 days after initiating paclitaxel administration, once thermal hyperalgesia had fully developed. i.p. Administration of rosuvastatin during 7 consecutive days induced a dose-dependent increase in the response latency in the hot plate (licking a paw or jumping off the plate), expressed as % change of the basal score before rosuvastatin (Figure 5A). The % Antihyperalgesia during the 14-day total period of testing amounted to 16.6 ± 3.9%, 28.8 ± 2.5%, 45.6 ± 5.7%, and 69.3 ± 4.6%, for doses of 3, 10, 30, and 100 mg/kg of rosuvastatin, respectively, all the % Antihyperalgesia scores being significantly higher than that obtained after vehicle administration (Figure 5B). The calculated ED_50_ value for rosuvastatin was 32.50 mg/kg with a 95% CI of 22.51 mg/kg to 46.91 mg/kg (Figure 5C).

Duloxetine administered i.p. during 7 consecutive days, induced a dose-dependent increase in the hot plate latency (Figure 5D). The % Antihyperalgesia during the whole 14 days of testing amounted to 20.9 ± 1.4%, 40.9 ± 3.3%, 40.9 ± 4.0%, and 57.4 ± 2.5%, for doses of 1, 3, 10, and 30 mg/kg of duloxetine, respectively, the % Antihyperalgesia scores being all significantly higher than that obtained after vehicle administration (Figure 5E). The calculated ED_50_ value for duloxetine was 15.53 mg/kg with a 95% CI of 6.72 mg/kg to 35.87 mg/kg (Figure 5F).

**Figure 6:**
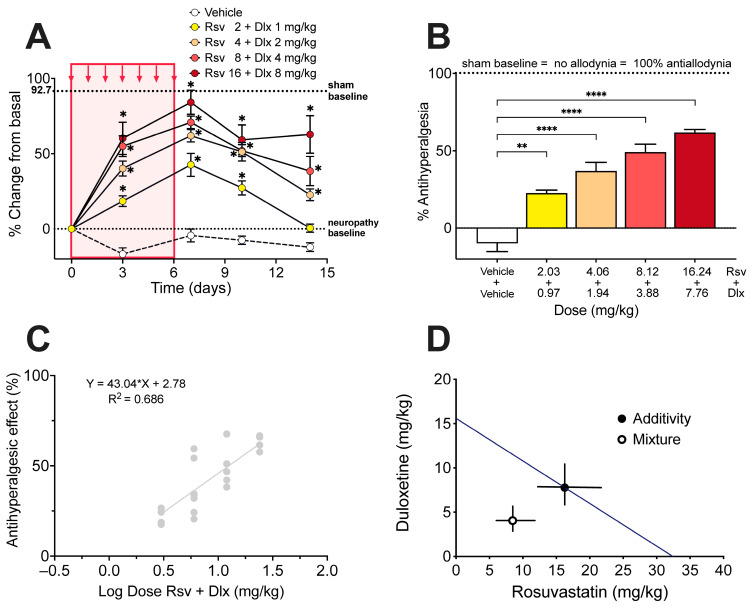
Effect of rosuvastatin/duloxetine combination on thermal hyperalgesia induced by paclitaxel in mice, and isobologram of the interaction. (**A**) Time course of the antihyperalgesic effect of vehicle and rosuvastatin (Rsv)/duloxetine (Dlx) combination administered in fixed ratios of their respective ED_50_, expressed as % change of hot plate scores (in g) regarding basal score at day 0 (i.e., 10 days after starting paclitaxel administration). Intragroup statistics: * *p* < 0.05, two-way ANOVA, F_time_ (3.127, 68.78) = 75.80. (**B**) % Antihyperalgesia after vehicle and rosuvastatin/duloxetine combination, calculated from area under curves of (**A**), considering 100% antihyperalgesia (i.e., no hyperalgesia) the sham control baseline (taken from Figure 1B). Statistics: ** *p* < 0.01, **** *p* < 0.0001, one-way ANOVA, F (4, 22) = 33.20. (**C**) Dose-response data representing the antihyperalgesic effect (in %) of rosuvastatin (Rsv)/duloxetine (Dlx) combination, expressed as dose logarithm. The equation for linear regression and the goodness of fit (R^2^) is shown in the graph. The ED_50_ of rosuvastatin/duloxetine combination was calculated from the regression line. (**D**) Isobologram of interaction for the antihyperalgesic effect of the rosuvastatin/duloxetine combination in mice with paclitaxel-induced neuropathy. The black circle on the straight line represents the point of theoretical additivity of the combination, whereas the white circle corresponds to the experimental point. The experimental point was significantly different from the theoretical point (mean ± SEM, *p* < 0.001), with an interaction index γ = 0.521, indicating superadditive interaction. The standard errors for rosuvastatin and duloxetine are resolved into rosuvastatin (abscissa scale) and duloxetine (ordinate scale) components and shown by horizontal and vertical bars, respectively.

From the ED_50_ of both rosuvastatin and duloxetine, the theoretical ED_50_ of the combination with a 95% confidence interval was calculated, and equieffective submultiples of them were established by mixing the drugs at fixed 1:1 ratio of their respective ED_50_ (1/2, 1/4, 1/8, and 1/16). The administration of the combination of rosuvastatin and duloxetine, in equieffective proportions of their respective ED_50_, induced a dose-dependent increase in the hot plate latency (Figure 6A). The % Antihyperalgesia for the total 14-day period of testing, showed that the association of rosuvastatin with duloxetine in fixed ratios of 1/2, 1/4, 1/8, and 1/16 of their respective ED_50_, produced % Antihyperalgesia scores of 22.6 ± 1.9%, 36.9 ± 5.5%, 49.1 ± 5.0%, and 61.8 ± 1.9%, respectively. All these antihyperalgesia scores were significantly higher than those obtained under vehicle administration (Figure 6B). The experimental ED_50_ for the rosuvastatin/duloxetine combination upon thermal hyperalgesia measured in the hot plate was 12.51 mg/kg with 95% CI from 8.95 mg/kg to 17.48 mg/kg (Figure 6C), which can be decomposed in 8.46 mg/kg rosuvastatin plus 4.04 mg/kg duloxetine. Isobolographic analysis for the administration of rosuvastatin/duloxetine combination showed that the experimental ED_50_ was significantly lower than the theoretical additive ED_50_, with an interaction index γ = 0.521, which means a superadditive effect of the combination upon hyperalgesia to heat induced by paclitaxel (Figure 6D).

### 2.4. Changes in Expression Levels of GFAP and CD11b Proteins in the Dorsal Horn Tissue of Mice with Paclitaxel-Induced Neuropathy, and the Effect of Rosuvasatatin/Duloxetine Combination Treatment

Expression levels of GFAP and CD11b proteins, as markers for astrocytic and microglial activation, respectively, was quantified by Western blot in dorsal horn tissue of mice with paclitaxel-induced neuropathy, following a similar antinociceptive drug treatment protocol to that used for algesimetric evaluation. Mice were daily injected with 2 mg/kg i.p. paclitaxel for 5 consecutive days, and 10 days after the first injection of paclitaxel, a 7-day antinociceptive treatment with rosuvastatin/duloxetine combination started at day 0. Mice with paclitaxel-induced neuropathy that did not receive the rosuvastatin/duloxetine combination served as controls for the effects of the rosuvastatin/duloxetine combination. The antinociceptive treatment consisted of daily i.p. injection of the ED_50_ dose of the rosuvastatin/duloxetine combination during 7 consecutive days, calculated from the regression line of dose-response data obtained upon von Frey testing (i.e., 9.40 mg/kg rosuvastatin plus 7.23 mg/kg duloxetine).

**Figure 7:**
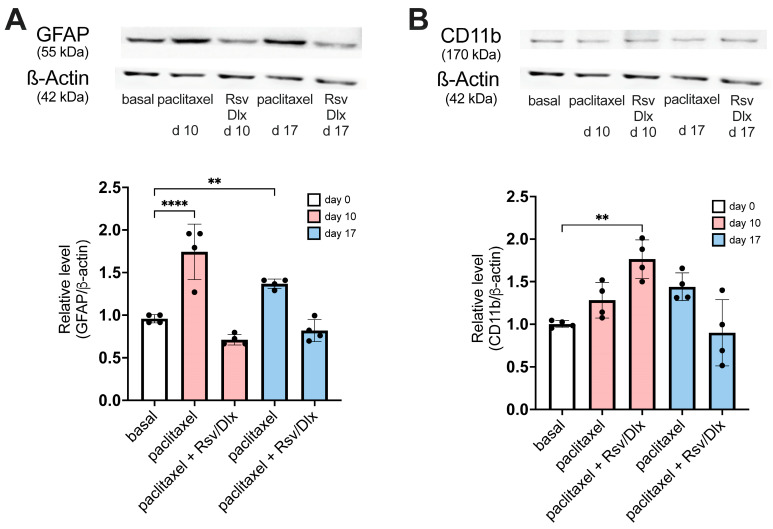
Expression levels of GFAP and CD11b proteins, assessed by Western blot, from dorsal horn tissue of mice with paclitaxel-induced neuropathy, and the effect of treatment with rosuvastatin/duloxetine combination. Paclitaxel 2 mg/kg was administered as i.p. injections for 5 consecutive days; 10 days after the first injection of paclitaxel, a 7-day antinociceptive treatment with rosuvastatin/duloxetine combination (at the ED_50_ dose calculated upon von Frey testing, containing 9.40 mg/kg rosuvastatin plus 7.23 mg/kg duloxetine, started at day 0. Mice with paclitaxel-induced neuropathy that did not receive the rosuvastatin/duloxetine combination served as controls. (**A**) Upper panel: Basal (day 0) and post-paclitaxel (days 10 and 17) blots of GFAP and β-actin in mice treated and non-treated with rosuvastatin/duloxetine (Rsv/Dulo) combination. Lower panel: The graph illustrates density data obtained from four blots after normalization to β-actin, expressed as fold-change relative to basal data (set as 1). GFAP expression was increased in paclitaxel-injected mice at day 10 (**** *p* < 0.0001) and day 17 (** *p* < 0.01) relative to basal score at day 0 (one-way ANOVA, F (4, 15) = 27.83). However, paclitaxel-injected mice treated with the rosuvastatin/duloxetine (Rsv/Dulo) combination did not show any statistical difference at day 10 and day 17 relative to basal score at day 0. (**B**) Upper panel: Basal (day 0) and post-paclitaxel (days 10 and 17) blots of CD11b and β-actin in mice treated and non-treated with rosuvastatin/duloxetine (Rsv/Dulo) combination. Lower panel: The graph illustrates density data obtained from four blots after normalization to β-actin, expressed as fold-change relative to basal data (set as 1). CD11b expression was not modified at day 10 or day 17 in paclitaxel-injected mice relative to basal score at day 0. However, the rosuvastatin/duloxetine (Rsv/Dulo) combination significantly increased (** *p* < 0.01) CD11b expression in paclitaxel-injected mice at day 10 (but not on day 17) relative to basal score at day 0 (one-way ANOVA, F (4, 15) = 8.831).

Figure 7A shows GFAP protein expression in the lumbar cord dorsal horn of mice with paclitaxel-induced neuropathy. At day 10, the dorsal horn of untreated neuropathic mice showed a significant almost two-fold increase in GFAP protein expression, as compared with basal values at time 0, while on day 17 the GFAP rise was reduced but remained significant. In contrast, the dorsal horn of neuropathic mice treated with the rosuvastatin/duloxetine combination showed normal levels of GFAP expression, both at day 10 and day 17 after starting the treatment.

Figure 7B shows that CD11b protein expression in the dorsal horn of mice with paclitaxel-induced neuropathy but without antinociceptive drug treatment is slightly increased on day 10 and day 21, but not significantly different from pre-paclitaxel basal CD11b protein levels. However, 7-day treatment with the rosuvastatin/duloxetine combination significantly (but transiently) increased microglial activation in mice with paclitaxel-induced neuropathy at day 10 after initiating the antinociceptive treatment, as related to basal level at time 0.

## 3. Discussion

We successfully replicated the model of peripheral neuropathy induced in mice by 5 consecutive daily injections of 2 mg/kg paclitaxel, as proposed by Nieto et al. [26], a model generating stable levels of mechanical allodynia and heat hyperalgesia as measured in the von Frey and hot plate tests. It should be noted that paclitaxel treatment did not impair motor coordination and balance in rotarod testing, as related to vehicle, ruling out a possible effect of paclitaxel in motor performance that may affect measurement of mice nociceptive behavior. Improvement in rotarod performance (increasing latency to fall) detected by intragroup statistics in paclitaxel and sham (vehicle injected) mice, represents a learned motor skill developed along successive trials, which was similar (no significant intergroup differences) in the two groups.

The main and most important result of the present study was that rosuvastatin and duloxetine, which individually are effective in some neuropathic pain models [16,17,18,19,20,21,22,28,29,30], interacted synergistically in mice with paclitaxel-induced neuropathy subjected to mechanical and thermal pain testing, which means that there was a potentiation of the antinociceptive effect of the drugs when given in combination. In fact, the ED_30_ of rosuvastatin or duloxetine alone upon von Frey testing were 12.98 and 7.10 mg/kg, respectively, while the equieffective dose of the rosuvastatin/duloxetine combination, i.e., the ED_30_ of the combination, only contained 3.02 mg/kg rosuvastatin and 1.65 mg/kg duloxetine. This means that the association of the drugs made it possible to reduce the dose of both drugs to less than a quarter when used to treat mechanical allodynia, at least in mice. Upon hot plate testing, the ED_50_ of rosuvastatin or duloxetine alone was 32.50 and 15.53 mg/kg, respectively, whereas the equieffective dose of the rosuvastatin/duloxetine combination contained 8.46 mg/kg rosuvastatin and 4.04 mg/kg duloxetine, meaning that the combination of the drugs again led to a reduction in the dose of each drug to near to a quarter, but this time in face to thermal hyperalgesia of the mice. If extrapolated to humans, this might be an important finding because the most common side-effects of duloxetine in clinical settings, such as fatigue, insomnia, anxiety, nausea, constipation, eye distention, and sexual dysfunction [31,32]—and particularly hypertensive urgency [33,34]—are somehow related to dosage and duration of treatment (for review of cardiovascular adverse events of duloxetine, see Park et al. [35]). In addition, it is important to consider that the combination of rosuvastatin with duloxetine would allow for reducing the dose of rosuvastatin and thus its associated side-effects. Indeed, the more relevant side-effect of this drug is statin-associated muscle symptoms (myalgia, myopathy, and myositis), which are the most well-documented and yet still do not have a unifying mechanism [36].

It seems worth remarking that the superadditive interaction between rosuvastatin and duloxetine, detected by isobolographic analysis upon von Frey and hot plate testing, originated from quite parallel regression lines obtained in the dose–response plots of the individual drugs, implicating that the potency ratio for these two drugs remained constant during testing of mechanical allodynia and heat hyperalgesia in neuropathic rat [24,37,38]. Theoretically, superadditivity in the effect of two simultaneously administered antinociceptive drugs signifies that the combined molecules act on anatomically and/or functionally different substrates for nociceptive processing, which may represent different neurons, different receptors in the same neuron, or even different sites of binding in the same receptor. In this regard, it is well known that duloxetine induces antinociception by inhibiting the reuptake of serotonin and norepinephrine in the spinal cord dorsal horn, thereby increasing the concentration of these neurotransmitters around spinal synapses established between primary afferents and second-order nociceptive neurons [10,39]. Furthermore, duloxetine has been proposed to have peripheral actions, as it can block voltage-gated sodium channels responsible for propagating action potentials primary nociceptive afferents [40,41], and to inhibit transient receptor potential channels of the TRPC5 subtype [42]. On the other hand, the antinociceptive activity of statins involves peripheral anti-inflammatory mechanisms as well as direct effects on nociceptive neurons and/or on some types of spinal glial cells in the central nervous system. Peripheral anti-inflammatory mechanisms of statins may include direct blockade of the mevalonate pathway, thereby disrupting several steps of the immune response, including immune cell migration, activation, signaling, and cytokine production [43,44]. On the other hand, once entering the central nervous system, statins could produce cholesterol-independent pleiotropic effects on neurons and glial cells by inhibition of mevalonate pathway/isoprenoid synthesis, affecting neurotransmitter levels, neurotransmitter receptors in the synapse, cellular viability, arborization of neuronal dendrites, and oligodendrocyte-mediated myelination (for reviews see [45,46]). The above data lead to the notion of quite different central and peripheral mechanisms of actions for duloxetine and rosuvastatin, which conceptually gives support to the present results showing a superadditive interaction between rosuvastatin and duloxetine. However, some degree of redundancy in the mechanisms triggered by rosuvastatin and duloxetine cannot be ruled out. For instance, statins can inhibit [47] or activate [48] signaling pathways downstream of some pain-sensing transient receptor potential channels, and interaction with the TRPC5 channel that detects cold pain has been recently described as part of the antinociceptive effect of duloxetine [42]. Thus, it is likely that the synergy of the rosuvastatin/duloxetine combination reported here arose from the interaction between the antinociceptive properties of rosuvastatin and duloxetine in yet undetermined molecular sites other than pain-detecting transient receptor potential channels.

As it is known, the von Frey test is the most widely used to determine tactile allodynia related to neuropathic pain in both rats and mice and is highly sensitive to peripheral changes in nociceptive processing such as those produced during characterization of non-steroidal anti-inflammatory drugs (for review see Yam et al. [49]). Instead, in the hot plate test, the animal responds to the thermal stimulus by licking or flicking its hind paw or jumping upward due to supraspinally integrated responses, and therefore, the hot plate test is more recommended to study the antihyperalgesic profile of centrally acting drugs [49]. On these grounds, the superadditive antinociceptive effect of the rosuvastatin/duloxetine combination in the von Frey test in mice with paclitaxel-induced neuropathy possibly represents a synergistic interaction mainly at the peripheral level of nociceptive pathways, while the superadditive effect of the combination upon hot plate testing might be viewed as a synergistic interaction mainly at the level of spinal integration of the antinociceptive response (e.g., serotonin- and/or norepinephrine-mediated spinal dorsal horn mechanisms, altogether with a presumably glial-dependent component). It should be noted, however, that the hot plate test used involves higher brain function and thus the result obtained is a supraspinally organized response; therefore, the results from hot plate testing cannot be explained only by changes in spinal dorsal horn nociceptive mechanisms (e.g., changes in GFAP and CD11b protein expression).

On the other hand, the fact that GFAP protein was overexpressed in the lumbar dorsal horn of mice with paclitaxel-induced neuropathy supports the involvement of astrocytes in allodynia and hyperalgesia observed in those animals. In contrast, a small but non-significant increase in the microglia marker CD11b was found in dorsal horn tissue. Although some studies have shown increased levels of microglial proinflammatory cytokines and chemokines in the spinal cord of paclitaxel-treated animals [13,50,51,52], other studies found no changes in cytokine levels at later stages of paclitaxel-induced neuropathy, during the allodynia phase, indicating that the alteration in these cytokine levels could have occurred during early development of neuropathy [53]. This finding correlates well with other observations suggesting activation of spinal astrocytes only, without significant microglia involvement [51,54,55], as occurred in the present study. Microtubule stabilizers such as paclitaxel can directly change the distribution and expression of intermediate filaments, including GFAP, in cultured astrocyte [56], and paclitaxel can be detected (although in low concentrations) in spinal cord after systemic treatment [57]. Thus, it seems possible that direct activation of astrocytes by paclitaxel may occur without the intervention of microglial cytokines, but rather as a result of downregulation of the glial glutamate transporters GLAST and GLT-1, which develops very rapidly in the spinal cord as soon as 4 h after the first administration of paclitaxel 2 mg/kg i.p. [55]. However, the possibility that activation of spinal cord astrocytes may be a secondary response to macrophage activation and neuronal damage after accumulation of macrophages in dorsal root ganglion cells has been amply debated in the literature (for a recent review, see Klein et al. [58]).

Results also showed that the rosuvastatin/duloxetine combination was able to reverse the GFAP protein overexpression in the lumbar dorsal horn of mice treated with paclitaxel. This is a rather expected result, because previous works have found that duloxetine alone downregulates increased levels of GFAP found in the spinal cord of mice with diabetic neuropathy [29]. Similarly, duloxetine in combination with pregabalin and/or tramadol also decreases GFAP levels in the spinal cord of mouse with chronic postischemic and spinal nerve ligation neuropathy [59]. Interestingly, simvastatin had been found to prevent overexpression of GFAP in the partial sciatic nerve injury mouse model of neuropathy [60], although a reversing (curative) effect was not addressed in that study. However, in the present study, it remains unclear which of the two drugs—rosuvastatin or duloxetine—was primarily responsible for downregulating the increased GFAP expression found in the dorsal horn of paclitaxel-treated mice, or if the drugs are only effective in combination. If GFAP levels in dorsal horn are really correlated with spinal cord disorders and neuropathic pain levels (for review see [61,62]), it can be hypothesized that the rosuvastatin/duloxetine combination is more effective than the individual drugs in lowering spinal GFAP levels, as in the present study the combination was more efficient against both mechanical allodynia and thermal hyperalgesia than each drug alone.

In contrast, the increase in CD11b protein expression levels induced by the rosuvastatin/duloxetine treatment in the dorsal horn of paclitaxel-treated mice was unexpected. Indeed, duloxetine administration has been found to downregulate the spinal expression of CD11b in a mouse model of diabetic neuropathy [29]. In addition, lovastatin intrathecally administered over lumbosacral spinal cord of rats attenuated the expression of CD11b in lumbar spinal cord dorsal horn of rats submitted to sciatic nerve injury [20]. The opposite observation found in the present experiments is difficult to reconcile with those previous results, even taking into consideration the different antibodies (polyclonal versus monoclonal anti-CD11b protein, from mouse versus from rat) and different statins used (rosuvastatin versus lovastatin), or the complex pleiotropic effect of the statins that might interact in various ways with duloxetine. However, mevastatin has been found to increase CD11b expression and to produce an amoeboid, macrophage-like microglial pattern in cultured slices of rat hippocampus [63], which somehow correlates with the present results. In addition, our result also correlates well with other data showing that three different statins strongly amplified pro-inflammatory cytokine protein and mRNA levels in microglia from adult rhesus monkeys, thereby exacerbating acute inflammatory reactions [64]. Thus, it remains to be clarified as to why the rosuvastatin/duloxetine combination gave rise to transient increased levels of CD11b (meaning microglial activation) in lumbar dorsal horn of paclitaxel-treated mice, altogether with the role that may be playing spinal microglia in paclitaxel-induced allodynia and hyperalgesia.

Although the mechanism underlying the ability of rosuvastatin to exert a synergistic action upon the duloxetine antinociceptive effect remains yet uncertain, this issue could constitute a potential basis for future clinical applications addressed to lower duloxetine and/or statin dosing together with a lowering of their side-effects. As mentioned elsewhere [65], due to scientific, practical, and ethical reasons, it is not possible to determine synergism in humans, and therefore, preclinical studies on drug combinations should be carried out in animals to obtain the basis and rationale for further studies in humans. Among current drugs for treating CIPN, although duloxetine is well positioned altogether with pregabaline, they have several side effects [32]. Drug combination strategies aimed to reduce duloxetine dosing—and therefore its side-effects—could be a promising therapeutic approach to optimize analgesia under CIPN conditions, provided the drug co-administered with duloxetine does not give rise to important side-effects by its own. To this end, statins probably represent the best alternative, since they currently are the gold standard therapy for hypercholesterolemia treatment due to their ease of dosing, limited drug interactions, and favorable safety profile. Although further studies are necessary to examine in detail the mechanism underlying the synergistic interaction between duloxetine and rosuvastatin, it can be concluded that this association could represent a potential therapeutic strategy aimed to treat some forms of chronic pain in humans such as CIPN, which deserves more investigation in clinical settings. Of note, other antidepressants with the same serotonin and noradrenaline reuptake inhibitor profile as duloxetine, such as milnacipran, should also be considered as a potential alternative for the treatment of CIPN, alone or in combination with a statin. Indeed, milnacipran is also effective in alleviating CIPN in mice [66], for review see [67] and therefore a likely candidate to develop synergism when combined with some statin.

In conclusion, the present results showed that in mice with paclitaxel-induced neuropathy, the rosuvastatin/duloxetine combination was more effective than the individual drugs in both algesimetric tests, giving rise to a superadditive antinociceptive effect against both mechanical allodynia and thermal hyperalgesia. Of note, the ED_30_ (von Frey testing) and the ED_50_ (hot plate testing) for the rosuvastatin/duloxetine combination contained about one-fourth of each drug than the ED_30/50_ of each drug individually. The 2-fold increase in spinal GFAP with no apparent change in the level of CD11b protein points to a role of astrocytes in paclitaxel-induced neuropathy. The reversal of overexpressed GFAP by the rosuvastatin/duloxetine combination suggests the antiallodynic and antihyperalgesic effects exerted by the drugs combination in some extent could depend on astrocyte inactivation. Extrapolated to clinical settings, the results suggest that statins could be useful in synergistically enhancing the efficacy of duloxetine in CIPN, potentially allowing for a reduction in the dose of duloxetine and its associated side effects, while achieving better analgesia.

## 4. Materials and Methods

### 4.1. Animals

Adult male CF-1 mice weighing 25–35 g, from the rearing facility of the Faculty of Medicine of the University of Chile, were used. They were maintained at room temperature (22 ± 2 °C) under a light–dark cycle of 12:12 h, and with free access to food and tap water. Before each experiment, the mice were acclimatized to the laboratory environment for at least 2 h. The experimental protocols were performed according to the Guide for the Care and Use of Laboratory Animals of NIH [68] and approved by the Animal Bioethics Committee of the Faculty of Medicine of the University of Chile (protocol CBA0737 FMUCH), following the ethical standards for the investigation of experimental pain in conscious animals [69]. Each experimental group consisted of 6 mice, a necessary number of mice that serves the purposes, as verified by the G* Power 3.1.9.7 software [70].

### 4.2. Materials and Drugs

The drugs used were paclitaxel (Fresenius Kabi, Santiago, Chile), rosuvastatin (Selleck Chemicals, Houston, TX, USA), and duloxetine (Tocris Bioscience, Minneapolis, MN, USA). Paclitaxel, dissolved in 50% Cremophor and 50% absolute ethanol (both from Sigma-Aldrich, St. Louis, MO, USA), was administered once a day for five consecutive days at a daily dose of 2 mg/kg i.p. Rosuvastatin was dissolved in 4% DMSO and 30% PEG-300 (both from Sigma-Aldrich) using double-distilled water, and administered once a day for seven consecutive days, at doses of 3, 10, 30, and 100 mg/kg i.p. Duloxetine was dissolved in 2% DMSO, 30% PEG-300, and double-distilled water, and administered once a day for seven consecutive days, at doses of 1, 3, 10, and 30 mg/kg i.p. All drugs were administered in a volume of 10 mL/kg, while controls groups received 10 mL/kg of the vehicle used to dissolve the respective drug.

The primary antibodies employed in the study were polyclonal anti-GFAP (1:10,000, rabbit, USBiological G2032-27J, United States Biological, Salem, MA, USA), polyclonal anti-CD11b (1:1000, rabbit, NB110-89474, Novus Biologicals, Centennial, CO, USA), and monoclonal anti-β-actin (1:2000, mouse, A5441, Sigma-Aldrich) that was employed to normalize the levels of protein detected. As secondary antibody, the goat polyclonal 115-035-174 antibody (Jackson InmunoResearch, West Grove, PA, USA) was used.

### 4.3. Measurement of Antinociceptive Activity

*Mechanical allodynia*: Mechanical allodynia was evaluated by means of the electronic von Frey test. After the animals were acclimatized for 30 min in a box of plexiglas with a wire-grid floor, a stimulus of increasing pressure was applied to the center of the plantar area of the right hind paw by means of an electronic von Frey filament (Dynamic Plantar Aesthesiometer, Ugo Basile, Italy) until the animal withdrew the stimulated limb. The force (in grams) required to achieve the withdrawal response was recorded, and an average of 3 consecutive measurements was defined as the mechanical threshold of the animal [71].

*Heat hyperalgesia*: Heat hyperalgesia was evaluated using the hot plate test. Each mouse was placed on a hot plate (Ugo Basile, Italy) at a temperature of 55 ± 0.5 °C, without movement restrictions, and the time (in s) in which the mouse presented a behavioral reaction to heat (lick the front or rear legs, jump off the plate) was measured, with a cut-off time of 20 s to prevent tissue damage [72]. The response latency of each mouse corresponded to an average of 3 consecutive measurements.

### 4.4. Measurement of Motor Coordination and Balance

To rule out a posible effect of paclitaxel in motor performance that may affect measurement of mice nociceptive behavior, the rotarod test was used according to the protocol of Dunham and Miya [73]. Animals were trained in at least two previous sessions. Each mouse was placed on the rod, which rotated at 20 rpm, and the time elapsed for animals fell off over a trial time of 60 s was measured. The time a given mouse spent on the rotating rod was measured three times and then averaged. As reported elsewhere, non-accelerating rotarods are better at detecting motor skill learning than accelerating rotarods [74].

### 4.5. Induction of Peripheral Neuropathy by Paclitaxel in Mice

Paclitaxel was administered at a concentration of 2 mg/kg i.p. in a volume of 10 mL/kg, for 5 consecutive days, as described by Nieto et al. [26], doses generating nociceptive neuropathy in mice with heat hyperalgesia and mechanical and cold allodynia. Healthy control sham mice received the vehicle employed to dissolve paclitaxel (see above) under the same administration schedule. The time course of evolution for the allodynia/hyperalgesia induced by paclitaxel was used to design the scheme of administration of rosuvastatin and duloxetine, as potential antinociceptive treatment for paclitaxel-induced neuropathy (see below).

### 4.6. Dose-Response Curves of Rosuvastatin and Duloxetine on the Paclitaxel-Induced Neuropathy

The antinociceptive effects of rosuvastatin and duloxetine (as well as the effects of the respective vehicles) in mice with paclitaxel-induced neuropathy were evaluated using the hot plate and the electronic von Frey tests. All algesimetric evaluations took place at noon, immediately before rosuvastatin, duloxetine, or vehicle administration. Dose-response curves were carried out using daily administration of rosuvastatin (3, 10, 30, 100 mg/kg i.p.) or duloxetine (1, 3, 10, 30 mg/kg i.p.) during 7 consecutive days, starting 10 days after the first administration of paclitaxel once stable levels of allodynia and hyperalgesia were observed. Dose ranges for rosuvastatin and duloxetine were taken from Miranda et al. [13] and from Bomholt et al. [75], respectively. Mice were evaluated on days 3, 7, 10, and 14, with day 0 being the day that rosuvastatin or duloxetine treatment (or vehicle control) was initiated. To construct time-course curves, values were expressed as % variation of scores after drug or vehicle (as compared to pre-drug/pre-vehicle scores) and plotted against time. To assess the global antinociceptive effect of the drugs, the areas under the curves were calculated using GraphPad Prism 9.0 software (GraphPad Software, Inc., San Diego, CA, USA), covering a period of time between the first day of injection of antinociceptive drug (day 0) and the last day of evaluation of the effects (day 14). The % reduction in area under curves, termed % Antiallodynia in the case of von Frey testing and % Antihyperalgesia in the case of hot plate testing, was calculated according to the equation 100 − [(A/B) × 100], where A is the area under the curve of a drug-treated animal and B is the mean area under the curve of the respective control sham group [76]. Plotting the % Antiallodynia and % Antihyperalgesia values against log dose allowed for obtaining the ED_X_ (i.e., the effective dose that produce X% of the maximal antiallodynic or antihyperalgesic effect) by linear regression analysis, the maximal antiallodynic or antihyperalgesic effect being the 100% Antiallodynia/Antihyperalgesia level (i.e., no allodynia/hyperalgesia) shown by the respective control group of sham mice. In the present study, the ED_50_ was used to assess the antihyperalgesic effect of rosuvastatin, duloxetine, or their combination on the hot plate test, while the ED_30_ was used to assess the effect of rosuvastatin, duloxetine, or their combination on mechanical allodynia, since the low sensitivity of the von Frey test against duloxetine and its combination with rosuvastatin did not allow reaching 50% antiallodynia, even with the highest doses used with these drugs. As reported elsewhere by Tallarida [37], concerning isobolographic analysis of drug interaction, the measured effect is very often 50% of the maximum (i.e., ED_50_), although any other level of effect reached by the drugs to be combined can be used. For example, Pelissier et al. [76] used ED_25_ when the algesimetric test was not very sensitive to a particular drug, either alone or in combination.

### 4.7. Isobolographic Analysis of the Interaction of Rosuvastatin with Duloxetine

The antinociceptive interaction of rosuvastatin with duloxetine in mice with paclitaxel-induced neuropathy was performed by isobolographic analysis, as described elsewhere [76,77,78]. The isobologram is a graphic method that involves calculating the theoretical additive dose of two drugs for producing a determined level of effect, and the statistical comparison with the combination dose that causes the same effect experimentally [24,37,38]. The combination of rosuvastatin with duloxetine was administered in a fixed 1:1 ratio of their respective ED_30_ (for von Frey testing) or ED_50_ (for hot plate testing), mixing equieffective submultiples of them (1/2, 1/4, 1/8, and 1/16). This procedure allowed to build a dose-response curve for the combination, from which the experimental ED_30_ or ED_50_ of the mixture can be calculated. The relation between the experimental value of the combination (experimental ED_30_ or ED_50_) and the theoretical value (theoretical additivity ED_30_ or ED_50_) in the isobologram determines the type of interaction, which can be expressed by the interaction index (γ = experimental ED_30/50_/theoretical additive ED_30/50_) between the drugs tested.

### 4.8. Proteins Determination in Spinal Cord: SDS-PAGE and Western Blott

Astrocytes were identified by the expression of GFAP that forms the intermediate filaments of the cytoskeleton, while the cells of the microglia were identified by the presence of the CD11b protein, a member of the β_2_ integrin family of adhesion molecules. The expression levels of these proteins, measured by the Western blot technique, were used to detect activation or inactivation of astrocytes and/or microglia in the dorsal horn of mice after induction of neuropathy with paclitaxel, and after treatment with the rosuvastatin/duloxetine combination. Animals were sacrificed by sodium pentobarbital overdose on days 0, 10, or 17 as related to the first injection of paclitaxel. The spinal cord was extracted into lumbosacral segments, washed 3 times in PBS, and immediately stored at a temperature of −80 °C. The tissues were homogenized in RIPA lysis buffer (1M TRIS-HCl pH 7.6, 150 mM NaCl, 5 mM EDTA, Triton X-100 1% *v*/*v*, 0.5% sodium deoxycholate, 0.1% SDS), and a cocktail of protease and phosphatase inhibitors (cOmplete™, Mini, EDTA-free Protease Inhibitor Cocktail Tablet, Sigma-Aldrich). The homogenate was centrifuged at 15,000 rpm for 20 min at 4 °C and the supernatant was collected for protein concentration determination using the bicincoinic acid technique (bicincoinic acid kit, Sigma-Aldrich).

For SDS/PAGE, 20 µg of total proteins was taken and mixed with loading buffer with blue bromophenol, heated at 95 °C for 5 min and loaded into 12% polyacrylamide gels (Mini-PROTEAN, Bio-Rad, Hercules, CA, USA). Electrophoresis was carried out at room temperature for 2 h at 80 V. The bands were electrotransferred to nitrocellulose membranes (trans-blot turbo mini-nitrocellulose transfer packs) using a Transwell equipment (Bio-Rad, Hercules, CA, USA) for 7 min. After incubating the membranes overnight at 4 °C with the appropriate antibodies (see above), they were rinsed with Tris buffer and incubated with the peroxidase-conjugated secondary antibody (see above). Bands were developed with luminol and revealed in a Chemiscope equipment (Clinx Science Instruments, China) adjusted to the best exposure time.

### 4.9. Data Analysis

Data are presented as means ± standard error of the mean (S.E.M.) and all statistical analyses, including calculation of area under curves and linear regression analyses, were performed with GraphPad Prism 9.5.1 software (GraphPad Software, Inc., San Diego, CA, USA). Comparisons of data between two groups was performed by two-tailed unpaired Student’s *t*-test. Comparisons involving three or more datasets (doses, times) were performed by one-way ANOVA, two-way ANOVA, or repeated measures two-way ANOVA (where appropriate), with Dunnett (intragroup comparisons against basal or vehicle scores) or Bonferroni (intergroup comparisons against corresponding scores in sham mice) post hoc test. Significance was accepted at a probability level of 0.05 or less.

## Data Availability

Not applicable.
